# Improving Hand-Grip Strength and Blood Pressure in Adults: Results From an AgingPLUS Pilot Study

**DOI:** 10.1093/geroni/igaa057.1966

**Published:** 2020-12-16

**Authors:** Abigail Nehrkorn-Bailey, Garrett Forsyth, Barry Braun, Kimberly Burke, Manfred Diehl

**Affiliations:** Colorado State University, Fort Collins, Colorado, United States

## Abstract

Based on adult inactivity, a new intervention named AgingPLUS was created, targeting motivational barriers to physical activity. Data come from a pilot study (N = 116), with 56 participants randomized to the AgingPLUS group (Mage = 63.52 years, SD = 7.89 years), and 60 randomized to the active control group (Mage = 63.06 years, SD = 8.30 years). Multi-group linear growth curve analyses examined improvements in hand-grip strength and blood pressure from pretest (Week 0) to immediate (Week 4) and delayed posttest (Week 8). Findings showed that only participants in the AgingPLUS group had significant improvements in hand-grip strength for the right (B = 1.34, p < .001) and left hand (B = 1.73, p < .001), as well as significant reductions in systolic (B = -3.28, p < .05) and diastolic blood pressure (B = -1.92, p < .01). These findings provide support for the efficacy of AgingPLUS.

